# Determinants of maternal and umbilical blood lead levels: a cross-sectional study, Mosul, Iraq

**DOI:** 10.1186/1756-0500-2-47

**Published:** 2009-03-24

**Authors:** Asma A Al-Jawadi, Zina WA Al-Mola, Raghad A Al-Jomard

**Affiliations:** 1 Department of Community Medicine, College of Medicine, University of Mosul, Mosul, Iraq; 2 Ninevah Directorate of Health, Mosul, Iraq; 3 Environmental Health Education & Resources Unit, College of Medicine, University of Mosul, Mosul, Iraq

## Abstract

**Background:**

The populations who are most sensitive to lead exposure from various sources are pregnant women and their newborns. Aiming to explore the presence of correlation between maternal and cord blood lead levels and to identify potential predictors that may influence both levels, the present study has been conducted.

**Methods:**

A cross-sectional study was conducted covering 350 full terms maternal-newborns pairs from Mosul maternity hospitals. Data were obtained directly from women just before delivery by the use of a detailed questionnaire form.

Maternal and umbilical blood lead levels were estimated using LEADCARE^® ^Blood Lead Testing System and Kits.

**Results:**

A positive significant correlation was found between maternal and cord blood lead values (r = 0.856, p = 0.001). By backward stepwise logistic regression analysis the followings emerged as significant potential predictors of high maternal blood lead: low parity, smoking and Hb level <11 gm/dl. Regarding cord blood lead: coffee consumption and high maternal blood lead were significant risk predictors. Milk and milk products consumption, calcium intake and low level of physical activity were significantly operational in the prevention of high maternal blood lead levels. Iron intake and also low level of physical activity were shown as significant protective variables against high cord blood lead values.

**Conclusion:**

Study results have provided baseline data needed to be transformed to decision makers to implement measures to eliminate lead from the environment and protect future generation from its deleterious effects.

## Background

There are numbers of studies published in developing countries that have evaluated maternal influences on umbilical blood lead levels (UBLLs) [1-4]. In Iraq very few studies were published covering population with assumed low exposure such as female in childbearing age and children [5,6]. The present study is the first report of a cross-sectional analysis of lead in maternal and newborn blood at the time of delivery in Mosul or probably in Iraq. It aims to determine the presence of correlation between maternal and umbilical BLLs among pregnant women attending Mosul maternity hospitals for delivery, and to identify potential predictors that may influence both levels.

## Methods

### Study population

Official permission was obtained from Ninevah Health Office and maternity hospitals administrations that were to be involved in this work. A written consent was taken from participants prior to the interview and blood sample collection.

Three maternity hospitals were chosen on the basis of having the largest monthly births and their accessibility for the whole population living in Mosul city. The present study adopted a cross-sectional study design among women who attended the delivery units in the three chosen hospitals. Data were obtained directly from the mothers themselves before delivery, (n = 370 maternal-fetus pairs).

The followings were the inclusion criteria for the participant:

(1) She is 15–49 years old.

(2) Mosul city resident for more than 3 years.

(3) Has a full term single viable pregnancy.

(4) Has no gestational diabetes or seizure.

(5) Has no psychiatric illness.

(6) Delivered by normal vaginal delivery.

Especially designed questionnaire form was used to collect information from the participants. This questionnaire has a high reliability (83.5%) and validity (82.1%). It included questions relating to maternal age, parity, coffee and tea consumption during the most recent month of pregnancy, milk and milk products consumption, smoking behavior, cosmetics uses and the degree of physical activity.

The last part of the form had questions concerning, history of chronic and acute diseases during current pregnancy & history of taking iron and calcium supplements. Hemoglobin level was taken from the case sheet.

Data collection was conducted between October 2006 and May 2007.

### Blood lead levels analysis

Analysis of blood lead was performed at the Environmental Health Education and Recourses unit of Mosul College of Medicine.

Blood Lead Levels (BLLs) were estimated by using LEADCARE^® ^Blood Lead Testing System and Lead Care Blood Lead Testing Kits by (ESA, Inc., USA). This system relied on electrochemistry and a unique sensor to detect lead in the whole blood. The contents of these kits are used specifically with LEADCARE^® ^Analyzer and Blood Lead Testing System.

Three ml of venous maternal blood samples were collected in lead free EDTA tubes and the same volume of umbilical cord blood was also collected immediately after birth from each corresponding newborn baby in EDTA tubes as well.

Fresh whole blood samples were thoroughly mixed in their containing EDTA tubes and accurately measured, 50 μL samples were transferred and mixed with treatment reagent until it turned brown. An exactly measured 50 μL blood mixture was then transferred to the kidney shaped active area of the sensor using the 50 μL pipette that was supplied with the LEADCARE^® ^System.

Having the sensor being properly placed into the sensor holder and its active area being thoroughly covered with the mixture, it was then pushed into the rest of the way into the sensor holder where the analyzer displayed the BLL in μg/dl after exactly 180 seconds. The range of the test is 1.4–65 μg/dl. "Hi" in the display window indicates that BLLs are greater than 65 μg/dl.

Analyses of refrigerated blood mixtures in the treatment reagent tubes were made in weekly batches. Mixtures were allowed to reach room temperature prior to analysis.

LEADCARE^® ^Blood Lead Controls were used to monitor the accuracy and precision of blood lead testing. They are prepared from bovine blood containing metabolized lead and they consist of a low level blood lead control; 6.4 ± 3.0 μg/dl (Level 1) and a high level blood lead control; 25.9 ± 4.0 μg/dl (Level 2).

Each control contains 2.0 ml lyophilized bovine whole blood that should be reconstituted with the provided 2.0 ml LEADCARE^® ^water with isothizolones (< 0.002%) as preservative. Reconstituted controls were used as would be a patient blood sample and as an internal quality control program.

### Statistical Methods

For the purpose of data analysis the cut off point used for both maternal blood lead levels (MBLLs) and UBLLs was ≥ 5 μg/dl [7]. Data were analyzed with a SPSS package version 13. Blood lead concentrations were log transformed due to non-normal distributions. Unpaired T-test was used to determine the presence or absence of significant differences between the two means. Pearsons' correlation was performed to find the degree of correlation between MBLLs & UBLLs. Backward stepwise logistic regression analysis was used in order to identify potential predicators contributing to elevated BLLs and UBLLs. P value of < 0.05 was considered significant throughout the present study.

## Results

The geometric mean (GM) of MBLLs at delivery was 3.26 ± 1.91 μg/dl with a range of 0.50–22.39 μg/dl, The GM of UBLLs was 2.29 ± 2.11 μg/dl and the range was 0.30–22.91 μg/dl. A highly significant difference was reported between the two GMs (p = 0.000).

Using untransformed data; 57 of pairs (16.3%) had an umbilical blood value higher than maternal blood lead.

It is worth noting that 5.4% of women had BLLs ≥ 10 μg/dl and 19.7%had a borderline value of 5–9 μg/dl. On the other hand 5.7% of newborns had BLLs ≥ 10 μg/dl and 7.7% had value falls within the range of 5–9 μg/dl.

**Figure 1:**
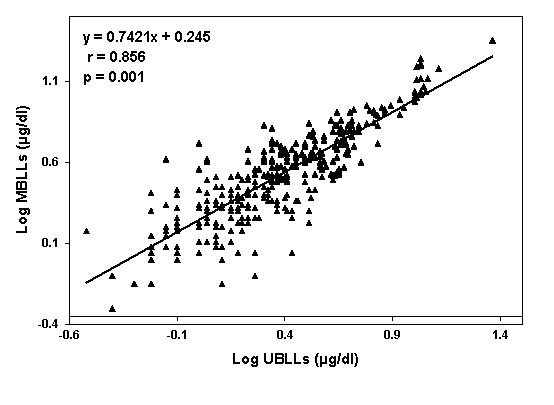
Correlation between MBLLs and UBLLs( µg/dl) after log transformation

Figure 1 exhibits a positive significant correlation between MBLLs and UBLLs (r = 0.856, p = 0.001).

The present study shows that women whose BLLs ≥ 5 μg/dl were more able to give newborns with an average BLLs significantly higher than those whose mothers' BLLs <5 μg/dl (5.48 ± 1.69 μg/dl versus 1.71 ± 1.74 μg/dl, p = 0.000)

[Additional file 1 STable 1] exhibits potential predictors that significantly increase or decrease MBLLs and UBLLs for ≥ 5 μg/dl. Regarding MBLLs the significant risk predictors are: low parity (p = 0.000), smoking (p = 0.049) and Hb level <11 gm/dl (p = 0.007), while low physical activity, milk and milk products consumption and calcium supplements intake during the current pregnancy played a significant protective role (p = 0.031, 0.000 and 0.000) respectively. On the other hand coffee consumption played a significant operational role in the development of UBLLs ≥ 5 μg/dl (p = 0.034). MBLLs ≥ 5 μg/dl played a very severe role shown by the value of Exp (β) = 150.070, and its degree of significance (p = 0.000). Low physical activity and iron supplements intake during the current pregnancy are shown as significant, protective variables (p = 0.000 and 0.045) respectively.

## Discussion

Blood lead in Iraq is not routinely measured in any health facility, therefore, there are limited data about the prevalence and predictors of high BLLs in both general population and high risk groups including women in child bearing age (pregnant & non pregnant) and children under five years of age [5,6]. Schnaas etal. [7] suggested that during pregnancy there is no threshold for the adverse consequences of lead on the subsequent intellectual child development.

The GM lead value for MBLLs and UBLLs reported in the present study are higher than those recorded by others [3,8,9]. This anticipated discrepancy is probably due to different locations, methods of lead levels estimation and governmental policies related to industry locations and phasing out the use of lead in house paints and gasoline. This suggests that the problem of leaded gasoline, in addition to uncontrolled distribution of electricity generators and battery maintenance shops are still the prime sources of lead exposure in Mosul City. Even though, car maintenance areas are present outside the city center, yet, there are a large number of houses present there [6].

In the present study MBLLs varied from 0.5–22.39 μg/dl and it is worth saying that 5.4% of the examined women had BLLs ≥ 10 μg/dl, moreover, 19.7% were with BLLs between 5–9 μg/dl which may be regarded as borderline value. Higher point prevalence (8.5%) was reported by Al-Naemi *etal.*[5] in the same venue.

Moline and Landerigan [10] reported that lead may cause neurological damage to the fetus at blood levels as low as 5–10 μg/dl. This problem is further compounded by lead mobilization from bony stores of the mother to enter fetal circulation [11].

In the present study the UBLLs varied between 0.3–22.91 μg/dl and 5.7% of newborns had a cord blood lead concentration levels ≥ 10 μg/dl, while 7.7% had borderline values (5–9 μg/dl). Deficits in cognitive and academic skills may occur at BLLs lower than 5 μg/dl, the lowest BLLs associated with adverse effect on these areas have not been adequately defined [12].

In the present study the GM of MBLLs is significantly higher than that of UBLLs (p = 0.000). This difference in GMs is in agreement with other studies [1,3,9,13,14].

In the present study UBLLs are positively correlated with MBLLs (r = 0.856, p = 0.001). This finding mimics results of other studies [1,3,4]. It seems, therefore, there is no protective barrier for the fetus from exposure to lead from its mother [15]. In fact, in the present study cord blood GM is 70.2% of that found in maternal blood. This suggestion is further supported by the finding of backward stepwise logistic regression analysis which showed that MBLLs ≥ 5 μg/dl are the strongest risk predictors of UBLLs ≥ 5 μg/dl (Exp (β) = 150.070, p = 0.000).

The present work proved that low parity is a significant risk predictor of development of high MBLLs (p = 0.000). Rothenberg *etal. *[16] stated different view where repeated pregnancy may affect the structure and blood flow of the placenta which results in more lead transfer to the fetus. In the current study smoking appears as a significant predictor of high MBLLs (p = 0.049). A survey carried out in Quebec City showed direct maternal exposure to smoke resulting in more lead in maternal blood and more lead transfer to the fetus [17]. However, in the present study smoking does not appear as a significant risk predictor of high UBLLs.

During this study Hb <11 g/dl emerged as a significant predictor for MBLLs ≥ 5 μg/dl (p = 0.007). The same conclusion was reached by Graziano *etal. *[18]. Health care system could reduce the prevalence of elevated BLLs by increasing efforts to prevent iron deficiency anemia in pregnant women. Fortunately, the present study showed that receiving iron supplements during current pregnancy has a significant protective effect against the development of high UBLLs (p = 0.045).

In the present study milk and milk products consumption and calcium supplements intake during the current pregnancy result in low MBLLs and UBLLs in a very highly significant way (p = 0.000 each). The same result was recorded in Mexico City [14]. This may be attributed to decrease lead absorption in the intestine or decreased maternal bone resorption with subsequent release of lead. Either mechanism could decrease MBLLs and potentially limit fetal accumulation of lead [19].

Low physical activity during pregnancy played a significant protective role against development of high MBLLs and UBLLs (p = 0.031 and p = 0.000) respectively. Harville *etal*. [9] in USA found different results. Over or under reporting of physical activity by study women could be attributed to this diverse result.

In the present study coffee consumption did not emerge as a predictor of high MBLLs, however it appears as significant risk predictor for UBLLs ≥ 5 μg/dl (p = 0.034). A similar result was found in USA [9]. This discrepancy in the results of the present study may be due to the fact coffee consumption is not a common habit in the Iraqi community.

### Methodological issues

Among the well known advantages of this study are the describing MBLLs and UBLLs and their burden on the study population, and determining their association with variables of interest. This finding can provide direction for potential area for more in deep future study.

An important point in this work is the large divers and city wide representative sample with a very high response rate (95.0%) although no information was gained about the non-respondents.

An important limitation in this study is the extent of over or under reporting of high BLLs' predictors which could not be determined.

Due to unavailability of atomic absorption spectroscope; BLLs were estimated by using LEADCARE^® ^Blood Lead Testing System by (ESA, Inc., USA). Although this system is mainly used for screening, but when it was used at a referral clinic/hospital versus the atomic absorption spectroscopy the two methods gave a correlation coefficient of 0.97 [20].

## Conclusion

For the first time in Iraq the present study provides a baseline data on MBLLs and UBLLs. Several local potential predictors emerged that determined BLLs.

These findings may be important to consider a prevention strategy. Iron and calcium supplementation could reduce maternal and newborns lead burden. Screening of women at childbearing age for the detection of elevated BLLs is mandatory. Furthermore, conducing studies to examine the effect of different BLLs on the development of children in the local community is a must.

## Abbreviations

BLLs: Blood Lead Levels; GM: Geometric Mean; MBLLs: Maternal Blood Lead Levels; UBLLs: Umbilical Blood Lead Levels

## Competing interests

The authors declare that they have no competing interests.

## Authors' contributions

All authors contributed in designing and conducting the present work, analyzing data, and drafting the manuscript. All authors read and approved the final manuscript.

## Supplementary Material

Additional file 1 The data provide results of backward stepwise logistic regression analysis for predictors of high BLLs among mothers and newborns. Click here for file
