# Supplementary material for: Determinants of maternal and umbilical blood lead levels: a cross-sectional study, Mosul, Iraq
Source: BMC Res Notes. 2009 Mar 24;2:47. doi: 10.1186/1756-0500-2-47 (PMC2663773; doi:10.1186/1756-0500-2-47)
Supplement: Additional file 1 — The data provide results of backward stepwise logistic regression analysis for predictors of high BLLs among mothers and newborns. [file 1756-0500-2-47-S1.doc]

Table (1) : Backward stepwise logistic regression model for MBLLs and UBLLs.

| **Variables** | **β** | **sig** | **Exp(β)** | **95% CI for Exp(β)** | |
| --- | --- | --- | --- | --- | --- |
| **MBLLs** |  |  |  |  | |
| Parity | 0.798 | 0.000 | 2.22 | 1.582 - | 3.119 |
| Physical activity | -0.450 | 0.031 | 0.638 | 0.424 - | 0.960 |
| Smoking | 0.710 | 0.049 | 2.034 | 1.003 - | 4.152 |
| Milk consumption | -0.818 | 0.000 | 0.441 | 0.320 - | 0.609 |
| Calcium intake | -1.753 | 0.000 | 0.173 | 0.079 - | 0.381 |
| Hb<11 gm/dl | 0.883 | 0.007 | 2.418 | 1.272 - | 4.594 |
| **UBLLs** |  |  |  |  |  |
| Physical activity | -1.647 | 0.000 | 0.193 | 0.122 - | 0.304 |
| Coffee consumption | 1.998 | 0.034 | 7.373 | 1.167 - | 46.580 |
| Iron intake | -0.828 | 0.045 | 0.437 | 0.194 - | 0.983 |
| MBLLs | 5.011 | 0.000 | 150.070 | 40.004 - | 162.967 |
